# Characterisation of a New Human Alveolar Macrophage-Like Cell Line (Daisy)

**DOI:** 10.1007/s00408-019-00288-3

**Published:** 2019-11-16

**Authors:** Laura R. Sadofsky, Yvette A. Hayman, Jesse Vance, Jorge L. Cervantes, Simon D. Fraser, Holly N. Wilkinson, James D. Williamson, Simon P. Hart, Alyn H. Morice

**Affiliations:** 1 Centre for Atherothrombosis and Metabolic Disease, Hull York Medical School, University of Hull, Cottingham Road Hull, Hull, HU6 7RX UK; 2 Respiratory Research Group, Hull York Medical School, University of Hull, Cottingham Road Hull, Hull, HU6 7RX UK; 3 Paul L. Foster School of Medicine, Texas Tech University Health Sciences Center, El Paso, TX USA

**Keywords:** Macrophage, Alveolar, THP-1, Daisy

## Abstract

**Purpose:**

There is currently no true macrophage cell line and in vitro experiments requiring these cells currently require mitogenic stimulation of a macrophage precursor cell line (THP-1) or ex vivo maturation of circulating primary monocytes. In this study, we characterise a human macrophage cell line, derived from THP-1 cells, and compare its phenotype to the THP-1 cells.

**Methods:**

THP-1 cells with and without mitogenic stimulation were compared to the newly derived macrophage-like cell line (Daisy) using microscopy, flow cytometry, phagocytosis assays, antigen binding assays and gene microarrays.

**Results:**

We show that the cell line grows predominantly in an adherent monolayer. A panel of antibodies were chosen to investigate the cell surface phenotype of these cells using flow cytometry. Daisy cells expressed more CD11c, CD80, CD163, CD169 and CD206, but less CD14 and CD11b compared with mitogen-stimulated THP-1 cells. Unlike stimulated THP-1 cells which were barely able to bind immune complexes, Daisy cells showed large amounts of immune complex binding. Finally, although not statistically significant, the phagocytic ability of Daisy cells was greater than mitogen-stimulated THP-1 cells, suggesting that the cell line is more similar to mature macrophages.

**Conclusions:**

The observed phenotype suggests that Daisy cells are a good model of human macrophages with a phenotype similar to human alveolar macrophages.

## Introduction

Macrophages are the predominant mononuclear phagocytes of the innate immune system and have a fundamental role in inflammatory processes throughout the body. Upon activation, macrophages are capable of eliciting a specialised immune response, the primary function of which is dictated by the stimulus and the environment in which the macrophage resides [1].

In vitro experiments requiring human macrophages use mitogenic (e.g. PMA) stimulation of a macrophage precursor cell line (e.g. THP-1 or U937), cells extracted during broncho-alveolar lavage (BAL) or ex vivo maturation of circulating primary monocytes. PMA stimulation activates protein kinase C resulting in phosphorylation of downstream signalling molecules [2]. This prolonged signalling activation regulates gene expression in a manner not comparable to human monocyte-derived macrophages [3].

The THP-1 cells are a human monocytic leukaemia cell line initially isolated by Tsuchiya et al. [4]. These cells grow in suspension and resemble human monocytes in morphology, secretory products, and membrane antigen expression [5]. A recent study by Forrester et al. has extensively characterised the cell surface markers of THP-1 monocytes and compared them to PMA-differentiated THP-1 macrophage cells [6]. Here we have derived a unique sub-clone of THP-1 cells which are capable of spontaneous and perpetual differentiation into alveolar macrophage-like cells without the need for PMA differentiation. This cell line, termed ‘Daisy’, was spontaneously derived [7] from a THP-1 clone sent as a gift. Upon initial culture, it was clear that they were not typical THP-1 cells since a large proportion of these cells were adherent. This phenomenon is not seen with THP-1 cells which normally grow in suspension. For this reason, we extensively characterised the new ‘Daisy’ sub-clone to investigate whether it retained key phenotypic and transcriptional features associated with mature macrophages, native THP-1 cells and/ or PMA-stimulated THP-1 cells.

## Materials and Methods

All cells were maintained in RPMI 1640 media (supplemented with 10% v/v Foetal Bovine Serum and 100 U/ml *Penicillin* and 0.1 mg/ml *Streptomycin* (Gibco, Paisley, UK)). THP-1 cells were differentiated using PMA (50 nM for 24 h, then 24 h PMA free), as described previously [8].

### Microscopy

Cells cultured in T25 flasks were visualised on an Olympus CK2 inverted microscope using phase contrast at 20 × magnification and captured on an Olympus C-5060 wide zoom digital camera. Transmission electron microscopy (TEM) was performed as described previously [8]. Briefly, cells were fixed (2.5% iso-osmotic glutaraldehyde in sodium cacodylate buffer, pH 7.3), post fixed (1% osmium tetroxide) then stained (1% uranyl acetate) before ethanol and propylene oxide dehydration. EPON resin embedded cells were then sectioned (Leitz UC6 ultra microtome) and visualised using a Jeol 2010 TEM.

### Mycoplasma Testing

THP-1 and Daisy cells were tested for mycoplasma infection using a MycoFluor™ mycoplasma detection kit (Molecular Probes, Paisley, UK) and the MycoProbe™ detection kit (R&D, Abingdon, UK) as per the manufacturers’ protocol.

### Assessment of Phagocytosis and Lipid Uptake

PMA/THP-1 and Daisy cells were incubated with zymosan beads and differentially stained, as described previously [8].

The ability of PMA/THP-1 and Daisy cells to take up unmodified lipid was assessed. Cells were incubated with 10% v/v Calogen (Nutricia, Wiltshire, UK) lipid rich liquid meal for a sub-optimal treatment time of 4 h before washing, staining with Oil Red O (ORO) and scoring according to the lipid-laden index (LLI) Colombo and Hallberg method [9]. Briefly, 100 cells were scored per experimental condition, assigning a value of 0–4 depending on the degree of lipid staining. The scores for the 100 cells were added to give the LLI. Cells from each well of a 24-well plate were scored in three independent experiments. The mean of the scores was then calculated and an unpaired, 2-tailed *t*-test was performed on the mean of the scores, for samples with unequal variance using Microsoft Excel 2010.

### Assessment of Immune Complex Binding

PMA/THP1 and Daisy cells were assessed for their ability to bind immune complexes. Fluorescent-labelled immune complexes were generated in-house as described by Hart et al. [10]. Mouse monoclonal FITC-conjugated anti-biotin antibody (170 ng/ml) was added to biotin labelled bovine serum albumin (BSA) (500 ng/ml) and the solution was placed on ice, in the dark (30 min) (BXB100).

Cells (1 × 10^5^) were then incubated with the fluorescent immune complex (BXB100) (100 ng/ml) for 30 min at 4 °C with gentle agitation. Cells were washed and re-suspended in FACSFlow Buffer (BD Biosciences) and analysed on a FACSAria II™ flow cytometer (BD Biosciences), running BD FACSDiva software (v6.1.3), counting 10,000 cells per sample. An unpaired, 2-tailed *t*-test was performed on the mean fluorescence, on separate cell combinations for samples with unequal variance using Microsoft Excel 2010 software.

### Cell Surface Immunophenotype

Cell surface Immunophenotyping of THP-1 cells, PMA/THP-1 cells and Daisy cells was assessed by flow cytometry as described previously [8] using a BD FACSAria II flow cytometer or using a BD Accuri C6 Flow cytometer and analysed using Flow-Jo software (v10). All antibodies were unconjugated mouse monoclonal (AbD Serotec, Oxford, UK) except CD32 [11]. A FITC-conjugated rabbit anti-mouse secondary was used for detection. Data were analysed with SPSS software (v19) and statistically significant differences in cell surface molecule expression were calculated. An independent samples Kruskal–Wallis test highlighted significant differences across all three cell types. Subsequent post hoc testing using Mann–Whitney tests then identified specific differences in expression.

### Gene Microarray

THP-1, PMA/THP-1 and Daisy cells were analysed for gene expression profiles, as described previously [8]. mRNA samples extracted using a NucleoSpin RNA II kit (Macherey–Nagel) were analysed using an Agilent G4851A SurePrint G3 Hmn GE 8 × 60 K Microarray on an Agilent G2505C microarray scanner at the University of York, Bioscience Technology Facility. Data were normalised by converting all the data values to log values (to base 2). Percentile shift normalisation and median baselining was performed on the data. A *t*-test was performed on each comparison group for each gene, and was corrected for multiple testing using the Benjamini–Hochberg FDR method. Principal component analysis (PCA) was performed on log2-transformed data using R v.3.6.1 [7]. Hierarchal clustering and Venn diagrams were based on differentially expressed genes above twofold different and over 5% alpha level significance. For hierarchal clustering, data were scaled, and the top 5000 most variable genes were clustered via Euclidian distance and Ward D2′s method within R v.3.6.1 [7]. Functional annotation was performed using the Database for Annotation, Visualisation and Integrated Discovery (DAVID) v.6.8 [12], with top annotations plotted against Benjamini–Hochberg FDR *P* value.

## Results

### Morphology of Daisy versus THP-1 cells by Light Microscopy

**Fig. 1:**
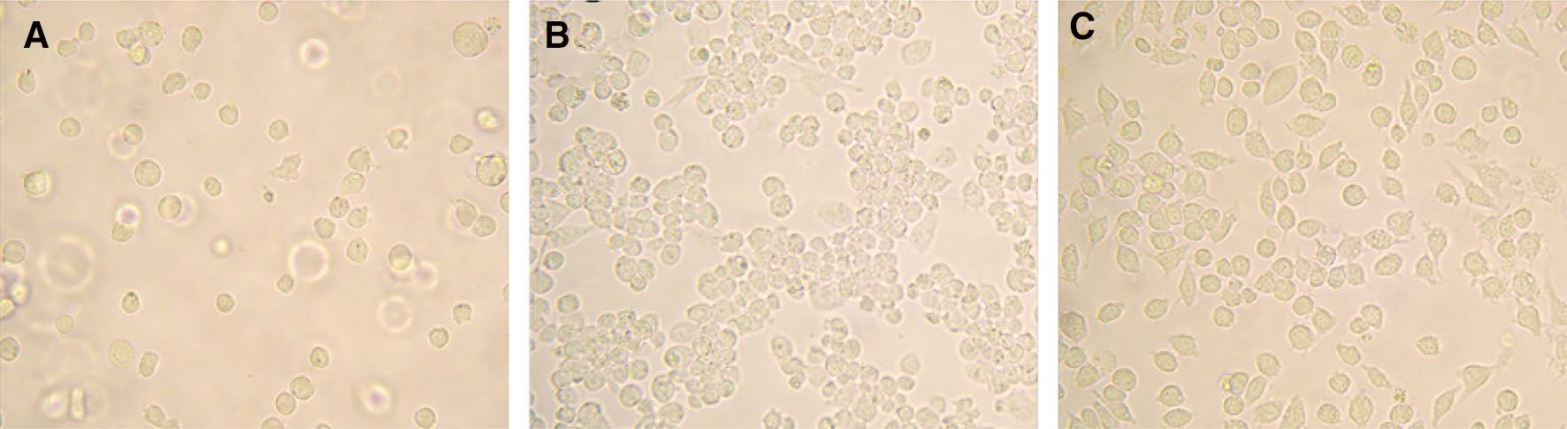
Morphology of daisy cells by light microscopy. THP-1 cells (**a**) appear predominantly suspended with some loosely adherent flattened cells making up no more than 5% of the total cells. When treated with 50 nM PMA for 24 h (**b**) and allowed 24 h recovery, THP-1 cells become adherent forming ‘clumps’ with increased cytoplasm and inhibited mitotic growth. Daisy cells (**c**) originally thought to be THP-1 cells show predominantly strongly adherent cells with a flattened morphology and pseudopodia without ‘clumping’

The morphology of the Daisy THP-1 sub-clone was compared with THP-1 and PMA/THP-1 cells by light microscopy (Fig. 1). THP-1 cells (Fig. 1a) grew predominantly in suspension and were not clumped with a small proportion of cells (<5%) very loosely adhering to the bottom of the tissue culture flask, becoming detached upon gentle agitation.

PMA/THP-1 (Fig. 1b) appeared slightly larger than THP-1 cells and were firmly adherent to the culture plate. These cells were clumped together and were flattened with some pseudopodia.

Daisy cells (Fig. 1c) appeared distinct. Although some cells grew in suspension and resembled native THP-1 cells, the majority formed an adherent cell monolayer. The adherent cells appeared larger and more flattened than the suspended cells, but did not clump together and show long pseudopodia, and in some cases appearing as long stretched out cells. When separated from adherent Daisy cells, non-adherent cells were capable of adhering to the new flask, indicative of a single population of cells. This Daisy phenotype appeared stable and has persisted for more than two years in two different research laboratories. All assays were done on the adherent population of cells.

### Mycoplasma Screening

Given the unexpected differences of the Daisy cells, Mycoplasma infection was screened using two separate methods. Neither the MycoFluor™ Mycoplasma Detection Kit (Molecular Probes) nor the colorimetric MycoProbe™ (R&D, Abingdon, UK) detection kit showed any Mycoplasma infection in the Daisy cells (data not shown).

### Morphology of Daisy versus PMA/THP-1 cells by TEM

**Fig. 2:**
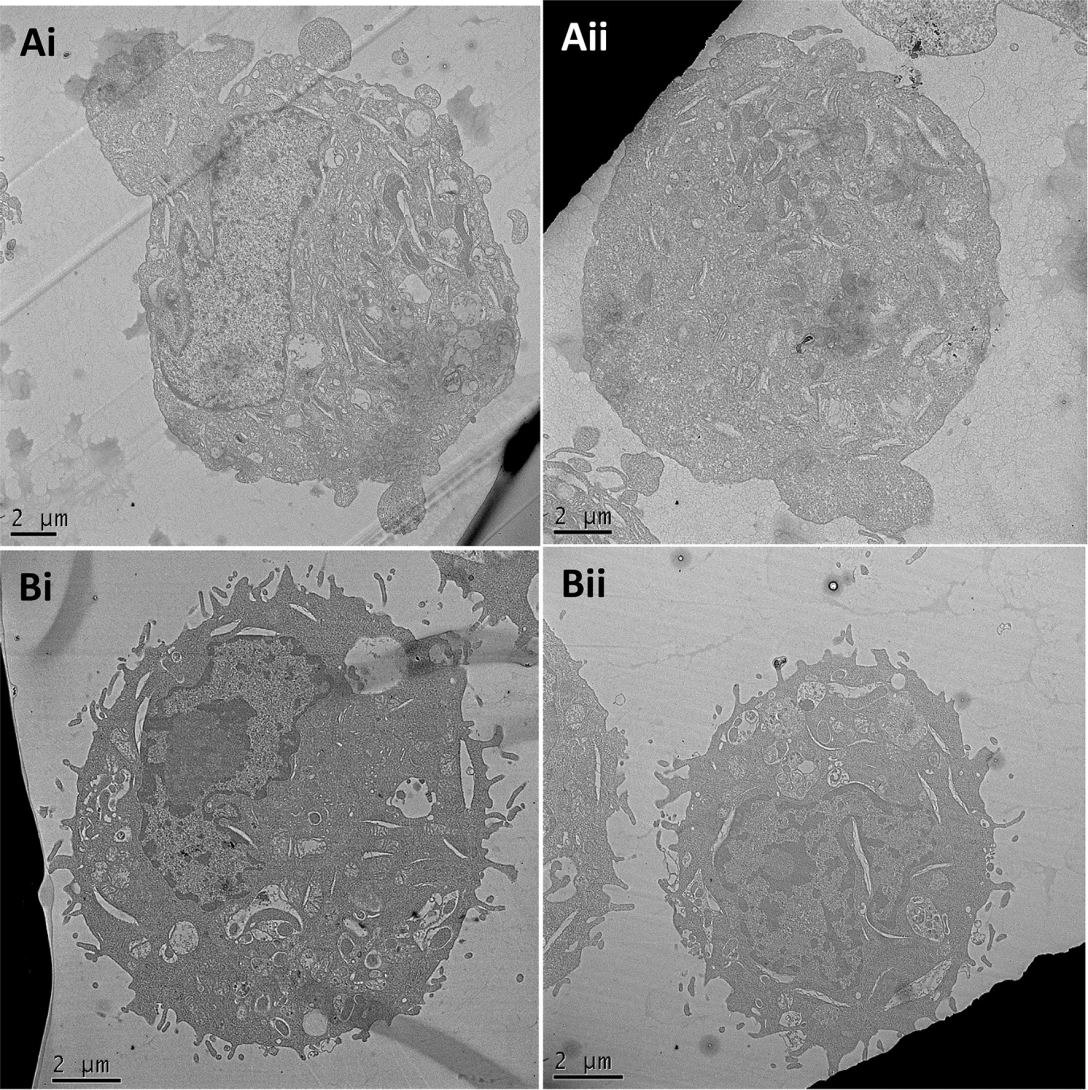
Morphology of Daisy cells by transmission electron microscopy. THP-1 cells (Ai and Aii) treated with PMA (50 nM; 24 h) appear approximately 14 µm in diameter with a large crescent-shaped nucleus containing euchromatin which is predominant. The cell membrane is slightly ruffled and there appears to be a number of vesicular inclusions and cholesterol clefts. Daisy cells (Bi and Bii) appear similar in size, shape and granularity; however, they show a higher proportion of heterochromatin, pseudopodia and vesicular inclusions. Pictures are representative images from 10 different cells for each cell type

Using TEM, Daisy and PMA/THP-1 cells appeared similar in size and shape, both with crescent nuclei (Fig. 2). The nuclei of the PMA/THP-1 cells (Fig. 2a) showed large areas of loosely coiled euchromatin, appearing as light grey areas, whilst Daisy cells (Fig. 2b) showed a high degree of tightly coiled heterochromatin, appearing as dark grey areas. The membrane of PMA/THP-1 cells showed some ruffling, whilst the Daisy cells showed a high degree of ruffling and pseudopodia. Both cells showed round vesicular inclusions within the cytoplasm and cholesterol clefts; however, they appeared to a higher degree in Daisy cells.

### Assessment of Phagocytosis

**Fig. 3:**
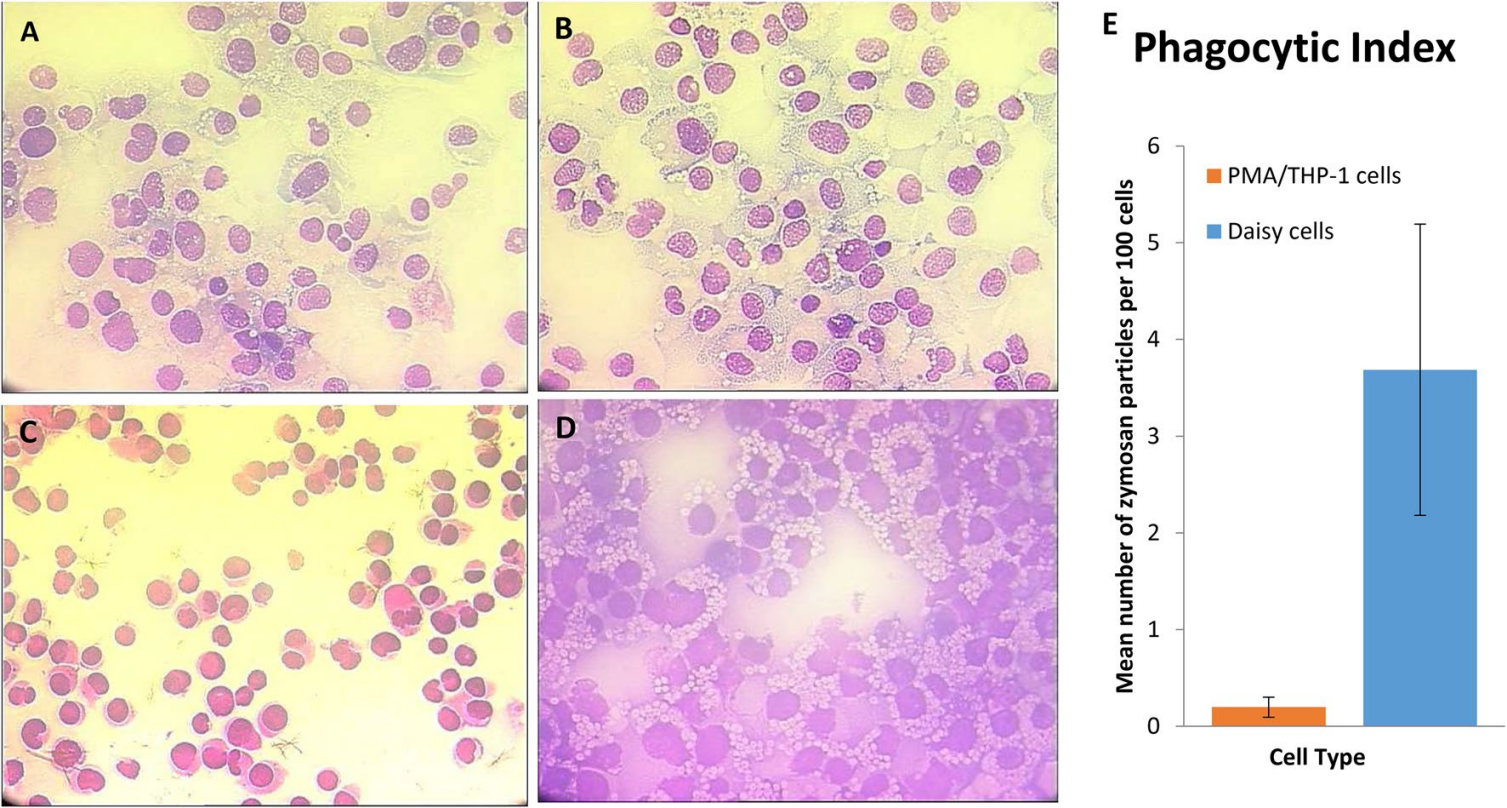
Assessment of Phagocytic capability of THP-1 and Daisy cells. PMA (50 nM; 24 h)-treated THP-1 cells (**a**) were differentially stained and compared to (**b**) THP-1 cells treated with PMA and zymosan (1 ng/ml; 1 h). **c** Untreated Daisy cells were also stained and compared with (**d**) Daisy cells treated with zymosan. Zymosan treated cells show phagocytosis of zymosan particles appearing as small purple dots surrounded by a white ring, approximately 3 µm in diameter at 40X magnification, contained within the cell cytoplasm. A phagocytic index was calculated (graph, right) by counting the number of zymosan particles within the cytoplasm of 100 cells and dividing this by 100 to give an average. Whilst THP-1 cells showed some phagocytosis, Daisy cells showed a marked, though not significant, increase in phagocytosis by comparison

The phagocytic ability of the cells was investigated by incubating with zymosan, differentially staining, and using a phagocytic index (Fig. 3). Whilst PMA/THP-1 cells showed some phagocytosis (Fig. 3b), a large number of cells showed no phagocytosis. Daisy cells showed a large number of cells containing many zymosan particles, although variation across three separate experiments was high (Fig. 3d) and no statistically significant difference was detected (Fig. 3e). The experiment shown in Fig. 3d demonstrates Daisy cells which have phagocytosed many particles represented by the large number of white dots in the cytoplasm.

### Assessment of Lipid Uptake

**Fig. 4:**
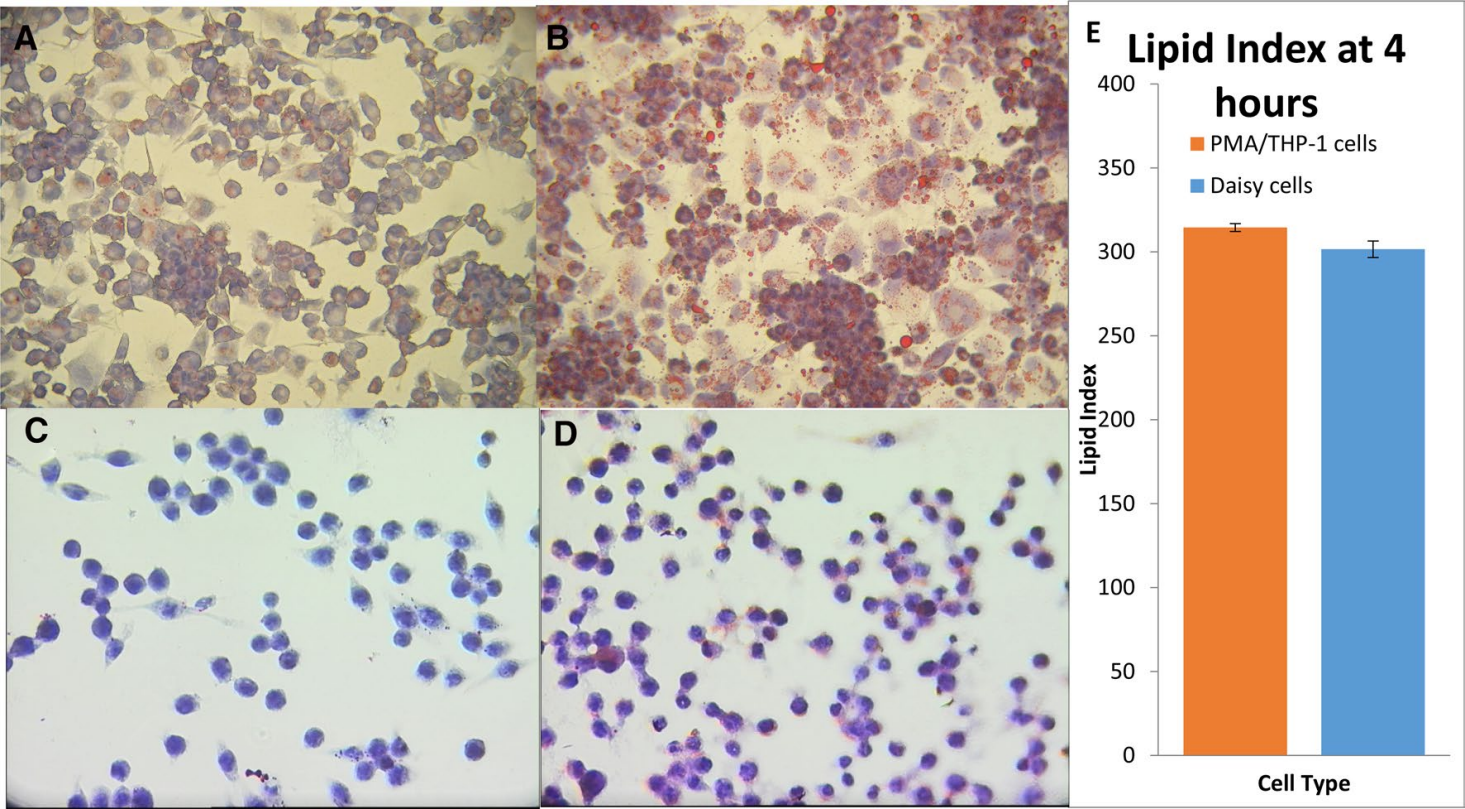
Assessment of lipid uptake of THP-1 and Daisy cells. PMA (50 nM; 24 h) treated THP-1 cells (**a**) were stained with ORO and hematoxylin and compared to cells treated with 10% v/v Calogen (**b**), a high fat liquid meal containing sunflower and canola oil, for 4 h. Untreated Daisy cells (**c**) were also stained and compared with Calogen treated cells (**d**). Slides were then scored and a mean was calculated across 3 × 24 slides for each cell type. Statistical analysis showed no significant difference in the amount of lipid accumulation between the two cell types

Lipid uptake by PMA/THP-1 cells and Daisy cells was detected with ORO. Slides were scored and a mean was calculated across 3 × 24 slides for each cell type (Fig. 4). Both cell types accumulated lipid after 4 h with the majority of cells showing between 50–75% lipid staining (Fig. 4b and d). No statistically significant difference was detected between the two cell types (Fig. 4e).

### Immune Complex Binding Capacity

**Fig. 5:**
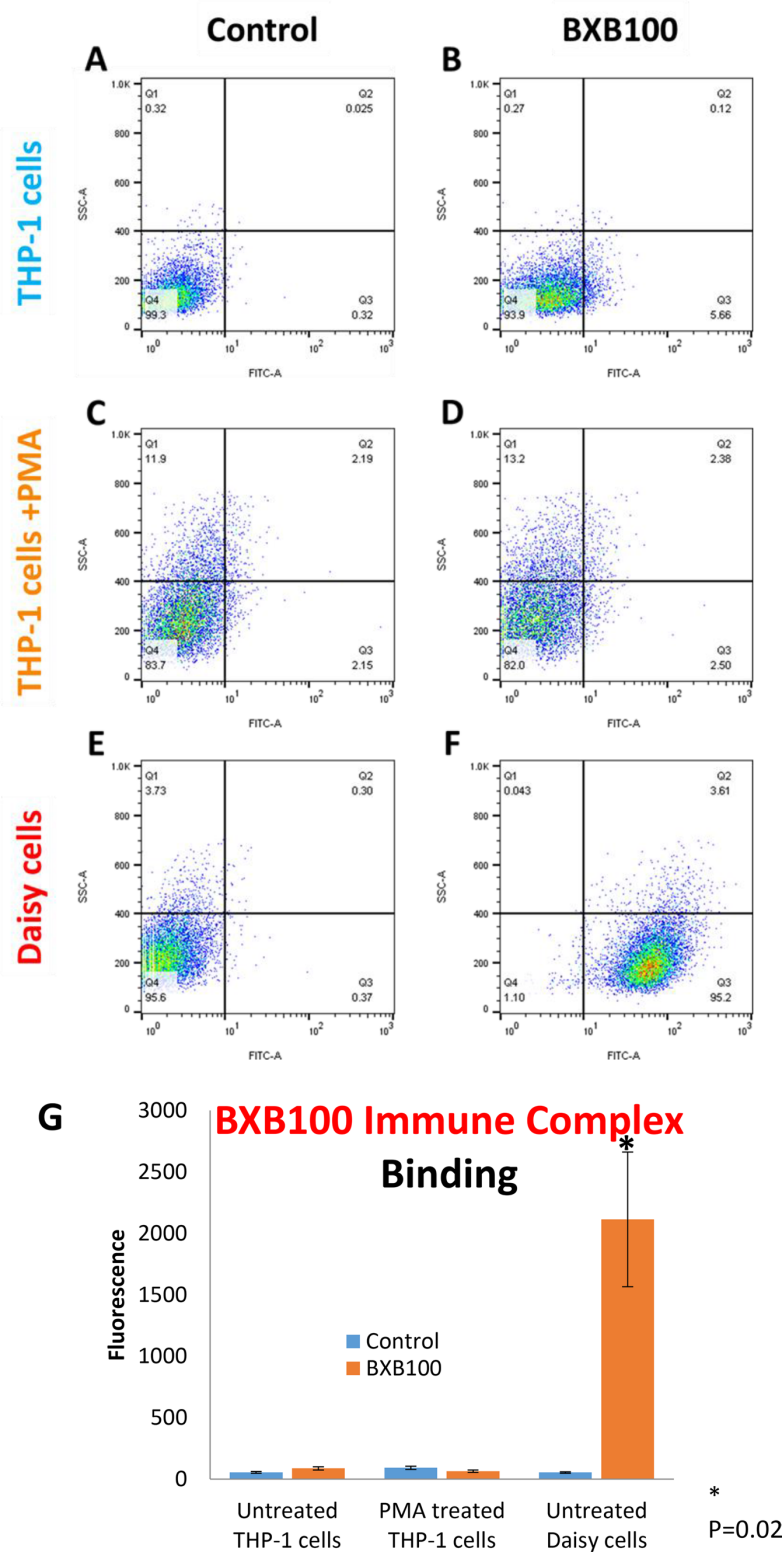
Immune complex binding capacity. THP-1 cells, PMA treated THP-1 cells and Daisy cells were incubated with fluorescently labelled BSA:Mouse IgG complex (BXB100) as a model for immune complexes. Representative pseudocolour plots displaying side scatter (SSC-A) and FITC fluorescence (FITC-A) of unstained controls (**a**, **c** and **e**) and following immune complex binding by THP-1, PMA/THP-1 and Daisy cells (**b**, **d** and **f** respectively). THP-1 cells (**a** and **b**), PMA treated THP-1 cells (**c** and **d**) and Daisy cells (**e** and **f**) are shown with appropriate quadrants (Q1–Q4) set to negative controls in THP-1 cells. Numbers shown in each quadrant represent percentage of cells. Q3 represents cells positive for the peptide. Plots are representative of *n* = 5. Bar chart representing the mean fluorescence with and without BXB100 treatment of THP-1, PMA treated THP-1 cells and Daisy cells (G). Significant to *p* = 0.02. Results are expressed as the mean ± SEM of five experiments

The ability of PMA/THP-1 and Daisy cells to bind immune complexes was compared using flow cytometry (Fig. 5). THP-1 and PMA/THP-1 cells bound very little immune complex, with mean fluorescence between 36–110 AU. Statistically no difference was seen between these two cell types. However, Daisy cells showed very high, statistically significant immune complex binding in the region of 1030–4010 AU (*p* = 0.02).

### Cell Surface Immunophenotype

**Fig. 6:**
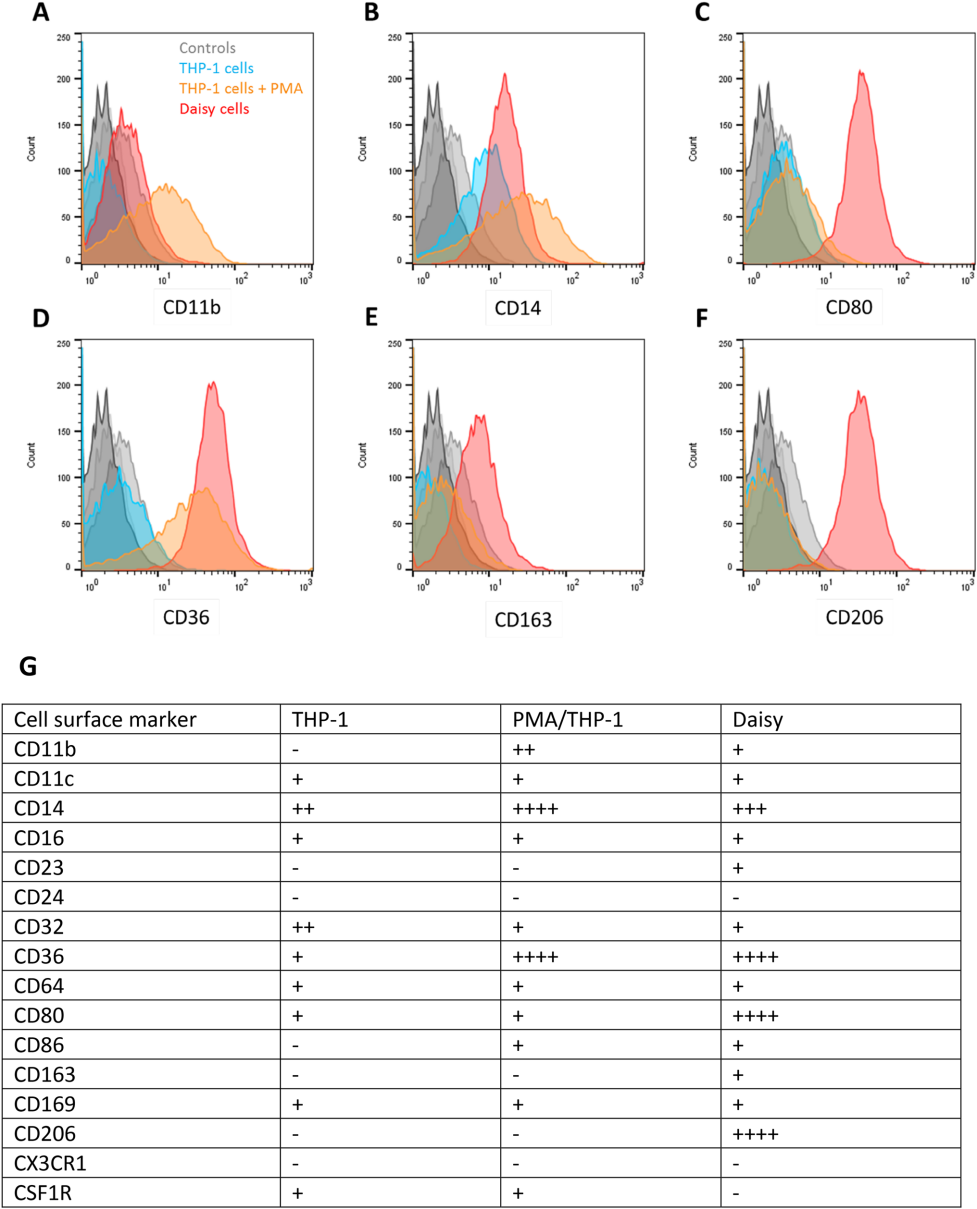
Representative histograms displaying selected markers on THP-1 cells, PMA treated THP-1 cells and Daisy cells. Levels of CD11b (**a**), CD14 (**b**), CD80 (**c**), CD36 (**d**), CD163 (**e**) and CD206 (**f**) were quantified by FITC fluorescence on conjugated secondary antibodies and assessed by flow cytometry. Unstained negative controls for THP-1 cells (white), PMA treated THP-1 cells (light grey) and Daisy cells (dark grey) are included. Staining for THP-1 cells (blue), PMA treated THP-1 cells (orange) and Daisy cells (red) are displayed. Histograms are representative of *n* = 5. (G) is a summary table comparing the phenotypes of the three cell preparations. Thresholds: − score equates to < the negative control (<100 fluorescence AU); + is fluorescence between 100 and 300 AU; +  + is fluorescence between 300 and 500 AU; +  +  + is fluorescence between 500 and 700 AU and +  +  +  + is fluorescence above 700 AU

Cell surface immunophenotype of THP-1, PMA/THP-1 and Daisy cells was compared using flow cytometry to assess expression levels of key markers (Fig. 6). All three cell types showed differing expression profiles. PMA/THP-1 cells showed significant increases in CD14, CD11b and CD36 with decreased CD32 expression compared with untreated THP-1 cells. Daisy cells showed decreased CD14 and CD11b with increased CD80, CD163 and CD206 expression compared with PMA/THP-1 cells.

We also observed expression of CD11c and CD169 in the Daisy cells but no significant expression of CX3CR1 or CSF1R (Fig. 6).

### Gene Microarray Data Analysis

**Fig. 7:**
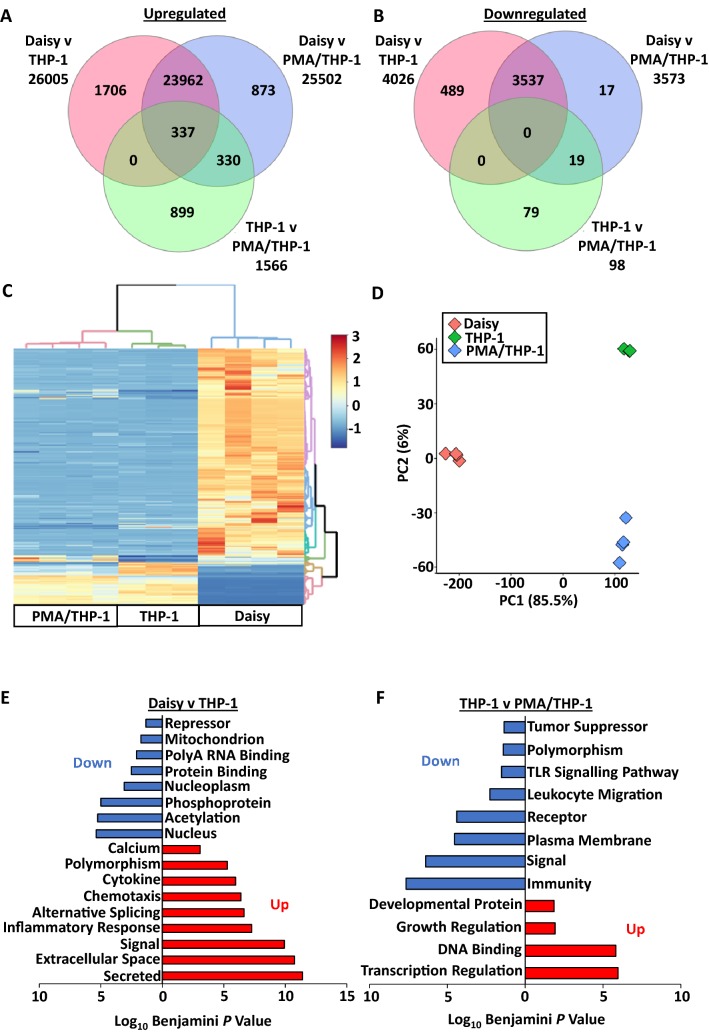
Venn diagrams and Hierarchal clustering analysis of differentially expressed genes in THP-1 cells, PMA-stimulated THP-1 cells and Daisy cells. Venn diagrams compare genes downregulated (**a**) or upregulated (**b**) in Daisy vs THP-1 cells (pink), Daisy vs PMA/THP-1 cells (blue) and THP-1 cells vs PMS/THP-1 cells (green). The data are filtered to only include fold change above 2 and *p* < 0.05. The heat map (**c**) demonstrates differential clustering of Daisy cells (*n* = 4), THP-1 cells (*n* = 3) and PMA/THP-1 cells (*n* = 4), based on the top 5000 most variable genes. Data were normalised (Z score) prior to hierarchal clustering analysis. Dendrograms coloured based on top clusters. Principal component analysis (**d**) confirms clustering of distinct groups. Functional annotation depicting pathways highly annotated in Daisy vs THP-1 cells (**e**) and THP-1 versus PMA/THP-1 cells (**f**). Up based on differentially expressed (DE) genes that are upregulated. Down based on DE genes that are downregulated

The magnitude of genetic difference between Daisy and THP-1 cells with or without PMA stimulation was analysed using an Agilent microarray system. Microarray data have been deposited in the ArrayExpress database at EMBL-EBI (www.ebi.ac.uk/arrayexpress) under accession number E-MTAB- 7169 and E-MTAB-4153. We have previously reported the change in gene expression in PMA/THP-1 compared to untreated THP-1 cells [8]. The difference in gene expression between Daisy and THP-1 or PMA/THP-1 cells was considerable (Fig. 7 a and b). 23,962 genes were upregulated and 3537 genes were downregulated in Daisy cells compared to both THP-1 cells and PMA/THP-1 cells. Smaller differences were observed in THP-1 comparison groups, where a total of 1566 genes were upregulated and 98 genes were downregulated in THP-1 cells versus PMA/THP-1 cells.

The differences between THP-1 and Daisy cells were even more apparent via hierarchal clustering analysis, where Daisy cells cluster were entirely independent of THP-1 groups (Fig. 7c). However, differences are also shown between the THP-1 cells and PMA/THP-1 cells, which cluster uniquely as shown by the heat map and PCA (Fig. 7c–d). Finally, functional annotation analysis of differentially expressed genes demonstrated over-representation of UniProtKB and gene ontology (GO) pathways associated with immune cells (Fig. 7e). For example, high enrichment was shown for inflammatory response, chemotaxis and cytokine in Daisy cells vs THP-1 cells. With respect to alternative splicing and polymorphisms, we note that these UniProtKB groups were significantly over-represented in Daisy cells vs THP-1 cells. While there were major differences between Daisy and THP-1 cells, we noted numerous functionally relevant UniProtKB and GO groups that were altered between THP-1 and PMA/THP-1 cells, including immunity, receptor and leukocyte migration (Fig. 7f).

## Discussion

We have characterised a unique sub-clone of THP-1 cells capable of spontaneous perpetual differentiation into macrophage-like cells termed ‘Daisy’. These cells are adherent and capable of phagocytosis of foreign material, lipid uptake and immune complex binding. They also express cell surface markers of human alveolar macrophages.

Morphologically, these cells are adherent in the absence of PMA stimulation, unlike THP-1 cells, indicating macrophage maturation. TEM revealed that they have a similar structural morphology to PMA/THP-1 cells, comparing well with published images of human macrophages [13]. High levels of euchromatin were seen in PMA/THP-1 cells suggesting increased DNA transcription processing. PMA stimulation is potent and long lasting, which may mask any subsequent activity during in vitro experiments. Daisy cells, however, showed less loosely coiled chromatin indicating that they are in a ‘resting’ state which is advantageous in the experimental setting.

A fundamental role of mature macrophages is to phagocytose foreign and damaged self-material. Both Daisy and PMA/THP-1 cells were capable of phagocytosis, with more particle uptake observed with the Daisy cells. However, no significant difference was seen between the two cell types. Both cell types were also equally capable of lipid accumulation, indicating cell maturity; macrophages are known to accumulate lipid in the airways [14] and diseases such as atherosclerosis.

The cells’ binding capacity was measured using immune complexes. Surprisingly, PMA/THP-1 cells were barely able to bind these particles despite expression of similar levels of all three IgG receptors (CD16, CD32 and CD64) to Daisy cells. Indeed, expression of a protein does not prove that it is functional, which may be the case here. This is in contrast to the work of Daigneault, et al. [15] who observed competent phagocytosis of opsonised latex beads by PMA/THP-1 cells. Daisy cells were able to bind large amounts of immune complexes indicating a significant difference between the two cell types and may suggest functional protein or binding by a different mechanism in these cells, for example, through complement receptors.

For cell surface phenotype characterisation, a panel of antibodies were chosen indicating myeloid cell origin (CD11b, CD14 and absence of CD24), cell maturity (CD16, CD23 and CD64), macrophage activation (CD163 and CD206), expression of co-stimulatory molecules (CD80 and CD86) and a known lipid receptor (CD36).

Daisy cells had lower CD11b, 14 and 32 expression than PMA/THP-1 cells, yet higher levels of CD36, 80, 163 and 206. The phenotype of the alveolar macrophage (AM) is known to vary greatly; however, expression of CD163, CD36, CD11c, and CD206 along with a lack of CD11b and CX3CR1 is compatible with the AMs phenotype [16-19]. A recent study by Mitsi et al.showed that the majority of AMs expressed high levels of CD206 and CD86 which supports our Daisy AM-like phenotype [20]. AMs help maintain immunological homoeostasis and host defence, while interstitial macrophages (IMs) appear to have a regulatory function within the lungs [21]. Reports have demonstrated differences between AM and IM in the lung [21], which may be distinguished by their unique combination of surface markers including CD206 [22]. The chemokine receptor CX3CR1, expressed in the mononuclear phagocyte system, including IM and alveolar DCs [23], is absent in AMs [24]. However, CD169 is absent in IM [25], but present in AM and in a new subset of CD169 + lung resident macrophages that are phenotypically and developmentally distinct from the AM or IM [26]. These can be clearly differentiated from AM as they are CD11c negative.

Daisy cells showed similar expression levels of CD11b, a major macrophage cell adhesion molecule, to THP-1 cells despite being adherent in contrast. Perhaps, CD11b then is not the major adherent molecule for these cells. Mature tissue macrophages downregulate CD11b and upregulate CD11c which could indicate a more mature macrophage phenotype of Daisy cells compared to PMA/THP-1 cells. Expression of the IgG receptors was similar across all cell types except THP-1 cells which showed higher expression of CD32. No CD24, the granulocyte adhesion molecule, was seen in any of the cells, which, coupled with CD14 expression, confirms the myeloid origins of Daisy cells. This molecule is only expressed by cells of the monocyte/macrophage lineage. A significant increase in CD36 was seen in PMA/THP-1 cells and Daisy cells. As expected, THP-1 cells showed no expression of markers of activation. PMA/THP-1 cells showed increased CD86, a co-stimulatory molecule required for anti-inflammatory T-cell differentiation, expressed by human macrophages when “classically activated” [27]. Daisy cells, however, showed high CD80 expression, a co-stimulatory molecule required for pro-inflammatory T-cell differentiation. Daisy cells also showed expression of CD163 and CD206, characteristic of alternative anti-inflammatory activation [27] not expressed by THP-1 cells whether stimulated or not.

Our data for cell surface phenotyping of THP-1 and PMA/THP-1 cells corresponds well with those from a recent paper by Forrester et al.[6]. They also showed an increase in CD36 and CD14 expression in PMA/THP-1 cells compared to THP-1 cells and CD64 was seen in both cell types. In agreement with our data, they also showed that CD32 was expressed to a higher degree in THP-1 cells compared to the PMA treated cells. Finally, they also found that neither THP-1 nor PMA/THP-1 cells expressed CD206, supporting our findings [6].

Gene array analysis corresponded well to the flow cytometry phenotyping results with increases in CD206 and CD80 gene expression seen in Daisy cells vs. THP-1 and PMA/THP-1 cells. In addition, Daisy cells had a decrease in CD11b and CD14 gene expression compared to PMA/THP-1 cells. The array data also demonstrated that Daisy cells were transcriptomically distinct from THP-1 cells, clustering entirely independently to THP-1 cells and PMA-stimulated THP-1 cells. A comparison between the transcriptome of Daisy cells and native macrophages or AM would be interesting for a future study. A study by Martinez however profiled the gene expression changes of human monocytes and macrophages [28]. It is difficult to compare the two studies and Martinez focused on polarisation of the macrophages; however, we did see downregulation of CCR2 and CX3CR1 genes in Daisy cells compared to THP-1 cells, indicative of mature macrophages [28].

Karyotyping of the Daisy cells versus the THP-1 cells was not done in this study. Investigations into the genomic aberrations of THP-1 cells has been performed in other studies and highlighted that these cells exist as a mixture of sub-clones and that these populations of cells can vary between labs [29, 30]. In future studies, it would therefore be interesting to determine whether the Daisy cells exist as a sub-clone of THP-1 cells due to genomic aberrations.

In conclusion, the genetically distinct Daisy cells display a macrophage-like morphology, function and phenotype. They are functionally mature and active in, most likely, an alternative fashion. These cells show markers of AMs, making them the first human AM-like cell line available. Such a cell line offers advantages over AMs obtained through invasive procedures like BAL, which renders limited cell, subject to individual and disease variability [31, 32]. The unique phenotype of AMs is determined by the lung environment [17] where GM-CSF is crucial for their phenotypic determination [33]. The Daisy cells have potential utility for future studies with the ability to spontaneously replicate and differentiate, reducing the need for blood donation in human macrophage research and eliminating PMA cell signalling interference.
